# Neighborhood Deprivation Is Strongly Associated with Participation in a Population-Based Health Check

**DOI:** 10.1371/journal.pone.0129819

**Published:** 2015-06-03

**Authors:** Anne Mette Bender, Ichiro Kawachi, Torben Jørgensen, Charlotta Pisinger

**Affiliations:** 1 Research Centre for Prevention and Health, Capital Region of Denmark, Glostrup, Denmark; 2 Harvard School of Public Health. Department of Social and Behavioral Sciences, Boston, Massachusetts, United States of America; 3 Faculty of Health Science, University of Copenhagen, Copenhagen, Denmark; 4 Faculty of Medicine, University of Aalborg, Aalborg, Denmark; Leibniz Institute for Prevention Research and Epidemiology (BIPS), GERMANY

## Abstract

**Background:**

We sought to examine whether neighborhood deprivation is associated with participation in a large population-based health check. Such analyses will help answer the question whether health checks, which are designed to meet the needs of residents in deprived neighborhoods, may increase participation and prove to be more effective in preventing disease. In Europe, no study has previously looked at the association between neighborhood deprivation and participation in a population-based health check.

**Methods:**

The study population comprised 12,768 persons invited for a health check including screening for ischemic heart disease and lifestyle counseling. The study population was randomly drawn from a population of 179,097 persons living in 73 neighborhoods in Denmark. Data on neighborhood deprivation (percentage with basic education, with low income and not in work) and individual socioeconomic position were retrieved from national administrative registers. Multilevel regression analyses with log links and binary distributions were conducted to obtain relative risks, intraclass correlation coefficients and proportional change in variance.

**Results:**

Large differences between neighborhoods existed in both deprivation levels and neighborhood health check participation rate (mean 53%; range 35-84%). In multilevel analyses adjusted for age and sex, higher levels of all three indicators of neighborhood deprivation and a deprivation score were associated with lower participation in a dose-response fashion. Persons living in the most deprived neighborhoods had up to 37% decreased probability of participating compared to those living in the least deprived neighborhoods. Inclusion of individual socioeconomic position in the model attenuated the neighborhood deprivation coefficients, but all except for income deprivation remained statistically significant.

**Conclusion:**

Neighborhood deprivation was associated with participation in a population-based health check in a dose-response manner, in which increasing neighborhood deprivation was associated with decreasing participation. This suggests the need to develop preventive health checks tailored to deprived neighborhoods.

## Introduction

The proportion of community-dwelling adults who agree to participate in population-based studies has declined rapidly in Western countries over the last decades. Typically, only about 50–60% accept participation in health checks including lifestyle interventions, depending on the content and design of the study [1], and even lower rates are seen for general health checks, e.g. in the English NHS health check [2]. A large number of scientific studies have demonstrated that participants tend to have higher education, higher income and more often are wage earners than non-participants [1,3]. Furthermore, most studies have found participants to be healthier than non-participants [4], with a marked lower mortality rate [5]. The uptakes of health checks are therefore inversely proportional to underlying need. If the aim is to improve population health through general health checks, the aim should be to increase participation rates among persons with the highest needs, that typically includes those with low socioeconomic position (SEP).

Neighborhood deprivation, measured as the proportion of residents with low income, low education and being unemployed, has been studied as a potential factor influencing participation in preventive screening programs [6]. Previous research has found that, even after adjusting for individual SEP, persons living in deprived neighborhoods exhibit a less favorable pattern of health behavior including smoking, being physically inactive and eating an unhealthy diet, compared to those living in more privileged neighborhoods [7,8]. Only one study examined the effects of neighborhood level SEP on participation in a general health screening program[9]. This study, conducted in South Korea, found that a composite deprivation index based on the proportion of unemployed, welfare beneficiaries, low housing quality, unskilled occupation and single-parent households was associated with decreased odds of participating in a general health screening program. Research focused on the impacts of neighborhood deprivation on participation in population-based cancer screening programs is also sparse and the associations depend on the specific cancer type [6,10]. Inconsistent results from Scandinavian countries exist on the association between neighborhood deprivation and risk of myocardial infarction [11] and mortality [12–16], and effect sizes in these countries seem to be of smaller magnitude when compared to less egalitarian countries [17]. As individuals have a tendency to cluster in neighborhoods with others with the same SEP it is common to adjust for individual SEP in multilevel analyses. However, at the same time neighborhoods influence the people that live in them [18]. Studies of neighborhood deprivation therefore help us to better understand barriers to participation, which are useful when designing effective population-based health checks.

In this paper we sought to examine whether neighborhood deprivation influences participation in a large population-based health check.

## Methods

### Study population and design

Inter99 is a population based randomized lifestyle intervention with a catchment area covering 73 census districts nested within 11 municipalities in the south-western part of the former Copenhagen County, Denmark. The study was approved by the local Ethics Committee (KA98155) and is registered at ClinicalTrials.gov (registration no. NCT00289237). The adult population (25–65 years) of this area comprised of 179,359 persons. This definition was chosen based on the assumption that most people above 25 years are active in the labor market, and the retirement age was set to 65 years in 1999. The study population (n = 61,301) was selected on December 2, 1998 as a sex and age stratified sample (selected age groups between 30 and 60 years) of all inhabitants in the source population. The design of the study has previously been described in detail [19]. At baseline, all persons in the intervention group (n = 13,016) were invited to a health check and assessment of risk of ischemic heart disease (IHD) at the Research Centre for Prevention and Health taking place between March 15, 1999 and January 31, 2001. They all had lifestyle counseling of varying intensity according to their assessed risk [19].

A total of 86 persons of the study population either emigrated or died in the period between date of randomization and baseline. Furthermore, between date of randomization and January 1, 1999, when data was retrieved from the registers a total of 77 persons moved to a municipality outside the study area and we were not able to identify the census district of 85 persons (1%), leaving 12,768 persons for analyses.

In Denmark each person is assigned a unique identification number at birth which enables citizens to be followed up for the rest of their life. Data can be used for research without person’s informed consent as long as predefined criteria are respected.

#### Individual level factors

Persons in the study population were categorized as participating (yes/no) if they attended the health check. Participation rate was defined as the proportion of invited persons within a neighborhood that attended the health check. Data on SEP was retrieved from national administrative registers administered by Statistics Denmark in the year before baseline [20]. Educational attainment was categorized into *basic* education (up to high school), *low* education (<2 years of vocational training), *middle* education (2–4 years of vocational training/education), and *high* education (>4 years; academic degree). There were 204 missing observations on educational attainment. When compared to the rest of the study population, a larger proportion of the persons with missing data on education were not in work and fewer participated in the intervention. However there were no clear differences in regards to prevalence of IHD, income, sex and age distribution (data not presented). There exists no automatic registration of immigrants’ education level which explains most of the missing data on education. Income (equalized disposable income) was calculated as the five year before baseline average household income after taxation and interest, divided by the number of equivalent adults in the household. Number of equalized adults in the household was calculated as follows: the first adult was given a weight of 1.0, each subsequent adult was given a weight of 0.5 and each child under 14 years was given a weight of 0.3. Employment status was categorized into *wage earners*, *retired* persons, and persons *not in work* (e.g. students, unemployed). Data on age and sex were retrieved from the Central Personal Registry.

#### Neighborhood deprivation

All persons between the age of 25 and 65 who by January 1, 1999 were living in the Inter99 study area (n = 179,097) were grouped into their respective census districts (n = 73), which were only established in 2006. Mean neighborhood population size was 4,315 persons (range: 710 to 8,889). Three variables on SEP were retrieved from national administrative registers on all inhabitants: income, which was defined as the mean family disposable income in 1999; employment status, which was dichotomized into wage earner and not in work on November 30, 1998; and educational attainment, which was assessed on the September 30, 1999 [21]. Neighborhood deprivation indicators were calculated as:

- Educational deprivation: Proportion of persons within each census district with *basic education (up to high school)*.
- Employment deprivation: Proportion of persons within each census district *not in work* (e.g. students, unemployed).
- Income deprivation: Proportion of persons within each census district belonging to the *lowest income quartile* (<24.150$/year) was used.

In order to prepare data for analyses, each of the three neighborhood deprivation indicators was ranked and grouped into quartiles: high, middle-high, middle-low and low level of educational, employment and income deprivation. A deprivation score (range 0 to 9) was calculated by summing the categorized variables (values from 0 to 3, with 3 being high deprivation); of each of the three neighborhood deprivation factors and was hereafter divided into quartiles.

### Statistical analyses

Descriptive statistics include mean participation rate in relation to proportion of inhabitants with basic education, proportion of persons not in work and proportion with low income. As there were no significant differences in participation according to neighborhood deprivation between men and women, all analyses were conducted for men and women combined.

We estimated the relative risks (RRs and 95%CI) of participating at baseline by conducting multilevel analyses with binomial distributions and log links; one model each for age, sex, each of the individual SEP factors, each of the neighborhood deprivation indicators and the deprivation score. Census district code and intercept was included in the random statement to account for intra-neighborhood correlation. The intraclass correlation coefficient (ICC) for was calculated as: $\frac{\sigma^{2}}{\sigma^{2}+1}$. We also calculated the percentage of proportional change in variance (PCV) as:$\;\frac{\sigma_{0}^{2}-\sigma_{1}^{2}}{\sigma_{0}^{2}}$ x 100. The $\sigma_{0}^{2}$ represents the variance of the initial model which only includes age and sex and $\sigma_{1}^{2}\;$ corresponds to the variance of a model with more variables. P-values for difference in participation between each category and the reference category were calculated together with a p-value for a dose-response relationship whenever relevant.

For each neighborhood deprivation indicator and the deprivation score separately the RR of participation was estimated while adjusting for age, sex, relevant other neighborhood deprivation factors and individual SEP factors. Confounders were identified based on a causal model. In four adjusted multilevel analyses, educational deprivation was adjusted for individual education, age and sex; employment deprivation was adjusted for educational deprivation, individual education, individual employment status, age and sex; and income deprivation was adjusted for educational deprivation, employment deprivation, individual education, individual employment status, individual income, age and sex. Finally, the deprivation score was adjusted for sex, age and all individual SEP factors. All analyses were performed using the statistical software program SAS (version 9.3; SAS Institute), and a p-value <0.05 was considered statistically significant.

## Results

### Descriptive statistics

The participation rate varied substantially between neighborhoods; participation ranged between 35% and 85% (mean = 53%).

**Fig 1:**
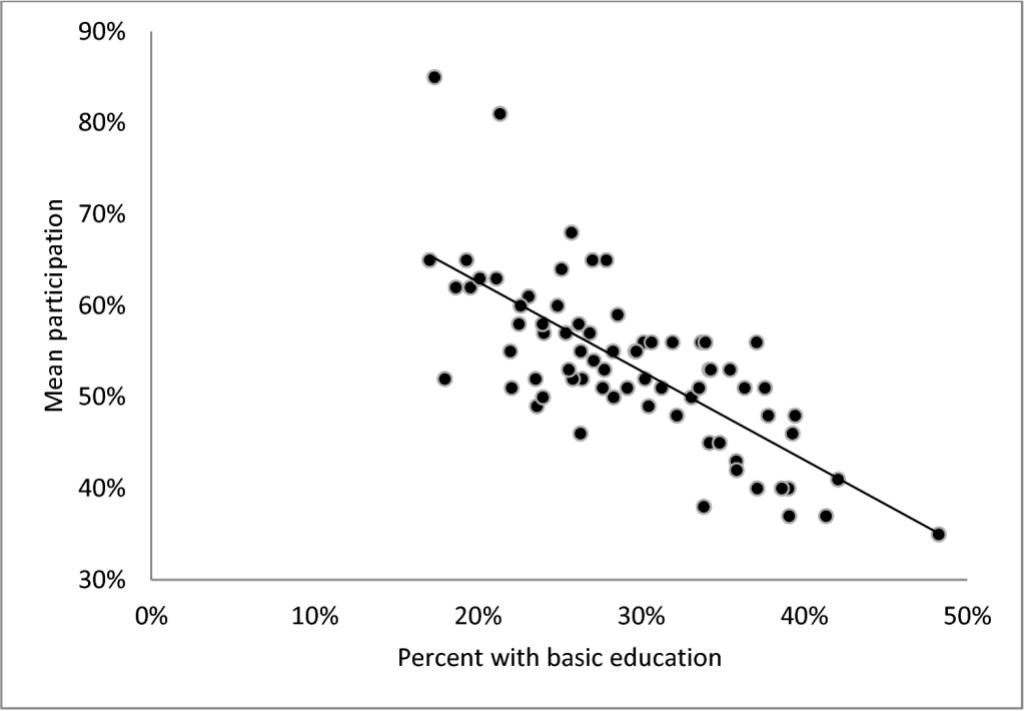
Mean neighborhood participation rate by percent with basic education in the neighborhood (neighborhoods n = 73, persons n = 12,768).

**Fig 2:**
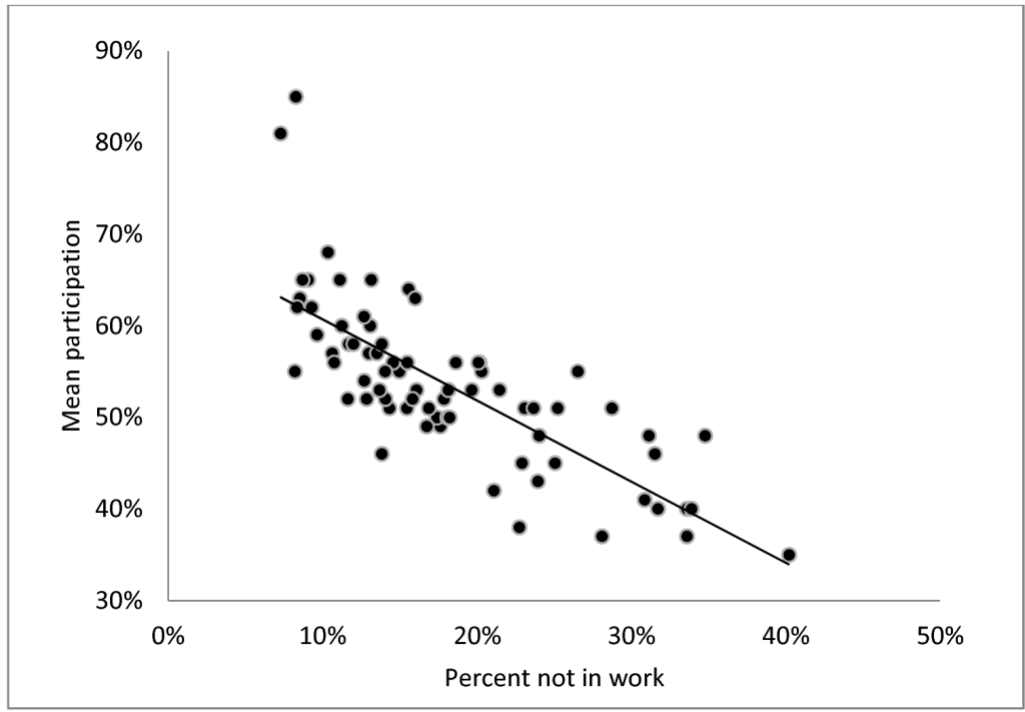
Mean neighborhood participation rate by percent not in work in the neighborhood (neighborhoods n = 73, persons n = 12,768).

**Fig 3:**
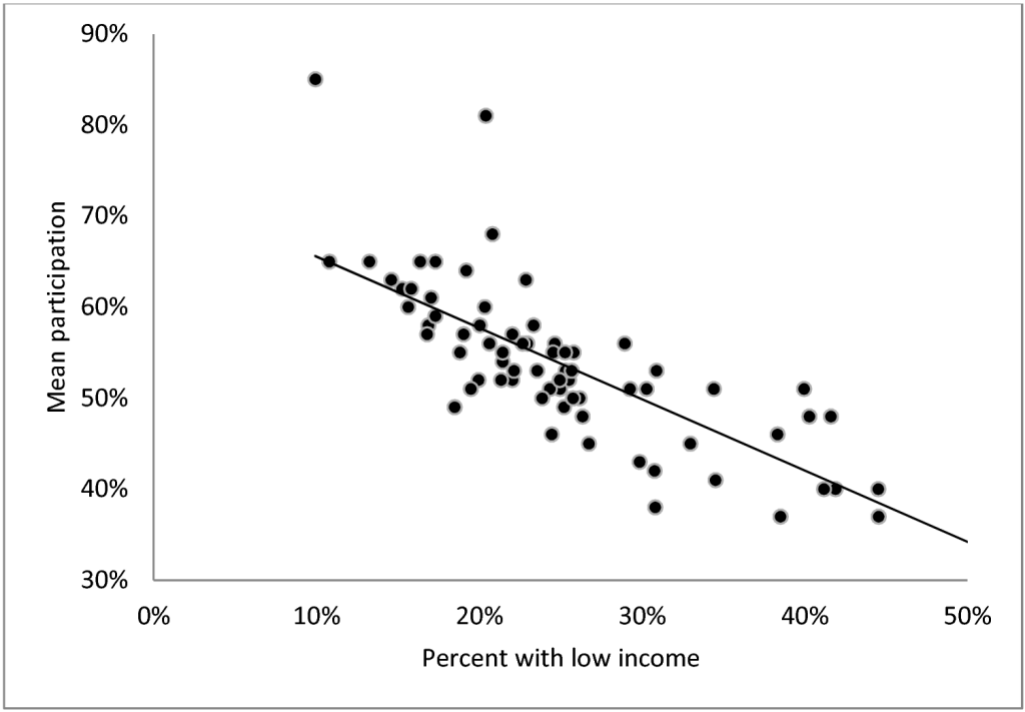
Mean neighborhood participation rate by percent with low income in the neighborhood (neighborhoods n = 73, persons n = 12,768).

Neighborhood deprivation likewise varied considerably between neighborhoods (Figs 1–3); on average 29% had basic education (range: 17% to 48%), 18% were not in work (range: 7% to 40%) and the percentage with low income ranged from 10% to 58% between neighborhoods. For all three neighborhood deprivation indicators we observed a dose-response association with mean neighborhood participation: increasing educational deprivation (proportion with basic education), increasing employment deprivation (proportion not in work) and increasing income deprivation (proportion with low income) were all associated with decreasing level of participation.

### Multi-level analyses

In multilevel models (Table 1) controlling only for age and sex we found increasing probability (relative risks [RR]s) of participation with increasing level of individual SEP and with decreasing level of each neighborhood deprivation indicator and the deprivation score. The largest differences in participation were seen for neighborhoods with different levels of income and employment deprivation and a composite deprivation score. Though there were large differences in participation according to neighborhood deprivation level, the ICC levels were modest. ICC levels of models including neighborhood deprivation in addition to sex and age were in general lower than those including individual SEP, sex and age. The PCV between the crude model (including age and sex) and the model including educational deprivation was 32% ((ICC_crude_-ICC_education_deprivation_)/ICC_crude_) and differences in neighborhood deprivation explained a somewhat larger part of the total variance in participation than did individual education (PCV = 16%). ICCs for the other neighborhood deprivation indicators and the deprivation score resembled that calculated for educational deprivation (Table 1).

**Table 1:** Multilevel models of association (RR, CI95% and P-value) between each individual- and neighborhood-level factor and participation, adjusted for sex and age.

Individual factors					Neighborhood level factors				
	RR	CI95%		P-value		RR	CI95%		P-value
Male (ref. = female)	0.96	0.93	0.99	**0.020**	Education deprivation				
ICC (SE)	11%		(0.017)		*Low*	1.32	1.22	1.43	**<0.001**
Age (years)					*Mid-low*	1.22	1.13	1.32	**<0.001**
* 30 or 35*	1	(ref.)			*Mid-high*	1.16	1.07	1.25	**<0.001**
* 40 or 45*	1.21	1.16	1.26	**<0.001**	*High*	1	(ref.)		
* 50*, *55 or 60*	1.17	1.11	1.22	**<0.001**	P-value for trend	***<0*.*001***			
P-value for trend	***<0*.*001***				ICC (SE)	8%		(0.002)	
ICC (SE)	11%		(0.017)		Employment deprivation				
Education					*Low*	1.37	1.28	1.47	**<0.001**
* High*	1.32	1.22	1.43	**<0.001**	*Mid-low*	1.23	1.15	1.32	**<0.001**
* Medium*	1.44	1.37	1.52	**<0.001**	*Mid-high*	1.15	1.07	1.23	**<0.001**
* Low*	1.28	1.23	1.34	**<0.001**	*High*	1	(ref.)		
* Basic*	1	(ref.)			P-value for trend	***<0*.*001***			
P-value for trend	***<0*.*001***				ICC (SE)	6%		(0.004)	
ICC (SE)	10%		(0.003)		Income deprivation				
Employment status					*Low*	1.37	1.28	1.47	**<0.001**
* Wage earner*	1.75	1.64	1.87	**<0.001**	*Mid-low*	1.28	1.19	1.37	**<0.001**
* Retired*	1.24	1.03	1.49	**0.022**	*Mid-high*	1.17	1.09	1.25	**<0.001**
* Out of workforce*	1	(ref.)			*High*	1	(ref.)		
ICC (SE)	8%		(0.002)		P-value for trend	***<0*.*001***			
Income					ICC (SE)	6%		(0.004)	
* I—Highest quartile*	1.62	1.54	1.72	**<0.001**	Deprivation score				
* II*	1.43	1.35	1.51	**<0.001**	*Low*	1.37	1.27	1.48	**<.0001**
* III*	1.30	1.23	1.37	**<0.001**	*Mid-low*	1.26	1.18	1.35	**<.0001**
* IV—Lowest quartile*	1	(ref.)			*Mid-high*	1.16	1.08	1.24	**<.0001**
P-value for trend	***<0*.*001***				*High*	1	(ref.)		
ICC (SE)	8%		(0.002)		P-value for trend	***<0*.*001***			
					ICC (SE)	7%		(0.005)	

RR relative risk; CI95% confidence interval; ICC intra class correlation coefficient; SE standard error

In adjusted multilevel analyses (Table 2), in which deprivation indicators were adjusted for individual SEP and neighborhood deprivation confounders in addition to sex and age, the coefficients for the deprivation indicators attenuated. Persons residing in the least deprived neighborhoods had, looking at the three deprivation indicators and the deprivation score, between 10–23% increased probability of participating, compared to those living in the most deprived areas. The strongest associations were found for educational deprivation and the deprivation score.

**Table 2:** Multilevel models of association (RR, CI95% and P-value) between neighborhood deprivation and participation; adjusted for relevant neighborhood deprivation and individual SEP confounders, sex and age.

	Education deprivation				Employment deprivation				Income deprivation				Deprivation score			
	RR	CI95%		P-value	RR	CI95%		P-value	RR	CI95%		P-value	RR	CI95%		P-value
*Low*	1.23	1.14	1.32	**<0.001**	1.17	1.08	1.28	**<0.001**	1.10	1.00	1.21	0.054	1.16	1.08	1.23	**<0.001**
*Mid-low*	1.16	1.08	1.25	**<0.001**	1.09	1.00	1.18	**0.044**	1.08	0.99	1.17	0.069	1.11	1.04	1.17	**<0.001**
*Mid-high*	1.11	1.03	1.20	**0.005**	1.06	0.99	1.14	0.091	1.05	0.98	1.13	0.141	1.06	1.00	1.13	0.065
*High*	1	(ref.)			1	(ref.)			1	(ref.)			1	(ref.)		
P-value for trend	***<0*.*001***				***<0*.*001***				*0*.*276*				***<0*.*001***			
ICC (SE)	7%		(0.002)		5%		(0.002)		NS				5%		(0.003)	

RR relative risk; CI95% confidence interval; SEP socioeconomic position; ICC intra class correlation coefficient; SE standard error, NS not significant

Though there was a tendency towards higher participation rate in the most affluent neighborhoods compared to the poorest neighborhoods, there was no overall trend in participation across income deprivation levels. In this model, when accounting simultaneously for other neighborhood deprivation indicators and individual SEP, the random effect of neighborhood became non-significant due to low intra-neighborhood variability. The random statement was therefore omitted in Table 2 for income deprivation.

## Discussion

Neighborhood deprivation—measured as a proportion of residents with no education, low-income families and persons not in work—was strongly associated with lower probability of participation in the Inter99 population-based health check. The effect measures were only slightly lower than associations between individual SEP and participation. Persons living in the least deprived neighborhoods had up to 37% increased probability of participating in the intervention compared to those in the most deprived neighborhoods. When individual SEP and neighborhood deprivation confounders were included in the multilevel models, the coefficients for neighborhood deprivation became attenuated, but all except for income deprivation nonetheless remained statistically significant. It appears that the variation in participation is explained both by differences in neighborhood deprivation and differences between individual SEP.

As no other European study has examined the effects of neighborhood deprivation on participation in a population-based health check, comparison is limited to one study from South Korea [9] and a recent review on effects of area level SEP on participation in cancer screening [6]. This review concluded that more studies are needed but, while the results remain mixed, studies on breast cancer demonstrate declining participation with increasing neighborhood deprivation [6]. Thus, our results are in line with both the studies on participation in breast cancer screening programs and with the results on participation in general health checks in South Korea [9] as well as neighborhood effects on health behavior [7,8].

Though we found a modest ICC in our sample, it was larger than ICCs reported from other studies of area level SEP and health outcomes [11–16,22]. This discrepancy may be ascribed to differences in outcome measures. It is likely that health behaviors including participation in a population-based health check are more influenced by neighborhood deprivation than chronic disease and mortality, as disease incidence depends on both genetic predisposition and risk behavior accumulated over a lifetime, while health behavior is defined in the moment. Second, most studies used municipality or parish level as the area variable, and these have on average more inhabitants and a larger variability with regards to both the number of inhabitants and SEP than do census districts.

The results of this paper bring new insights to the evidence on the linkage between neighborhood deprivation and health behaviors. To the extent that participation in a health check serves as a marker for health maintenance behaviors, our results illustrate an instance of the “inverse care law”, i.e. the uptake of health promotion is inversely proportional to underlying need [23]. A potential mechanism for our finding is the variation in social cohesion across neighborhoods. Kawachi et al. have described how neighborhood cohesion is crucial for the diffusion of innovations [17]. We hypothesize that residents of deprived neighborhoods may be disconnected from sources of information, or lack the social reinforcement and social support to participate in a health check. They may also be less trusting of public health authorities.

A limitation of this study is the missing data on census district resulting in the exclusion of 1% (n = 85) of the original study population as well as missing data on educational attainment excluding a further 2% (n = 204) in models including this variable. As census areas are based on 2006 data, changes in road names during the 7 year period from study start could be an explanation for the missing data on census district. In all of the 11 municipalities there were persons with missing census districts, supporting this assumption. Another weakness is the cross-sectional study design in which measures of educational and income deprivation are measured after participation. We however find reverse causality very unlikely, both as neighborhood deprivation changes slowly over time and as it is hard to think of a way in which participation in the health check should influence neighborhood educational and income deprivation. Individual SEP and employment deprivation on the other hand were measured before baseline, which ensures the temporal nature of the exposure*-*outcome association which is required in order to draw causal inferences.

Using census districts as the neighborhood measure has several strengths. The borders of the districts are typically based on boundaries following the physical division of major roads, division of urban and rural areas and following borders of housing associations. Furthermore, they are in many cases equivalent to school districts that represent small communities distinct from one another. The relatively small size of the neighborhoods minimizes dilution of neighborhood effects. Other strengths of this paper are the very large size of this population-based study, and the use of national register data for measures of individual SEP and neighborhood deprivation. The registers gave us the unique possibility to access objective data for all persons in the study population: both participants and non-participants. The Danish national registers have high quality and validity [20].

Whether to base our final conclusions on unadjusted models (neighborhood deprivation) or adjusted models (neighborhood deprivation + individual SEP) depends on whether individual SEP is considered a confounder or a mediator. On the one hand individuals may select their place of residence based on their predisposition to certain behaviors [24]. For instance, a person not in work may move to a neighborhood with a high proportion of other individuals not in work as these areas offer cheaper housing. On the other hand persons living in areas with a high percentage of persons not in work may have difficulties getting a job. Individual SEP may therefore simultaneously confound and mediate the effects of neighborhood deprivation on participation in a population-based health check.

In the future, tailoring preventive health checks to meet the needs of deprived neighborhoods may prove to be more effective in preventing disease at the population level than general health checks. Preventive health checks tailored to deprived neighborhoods in terms of poverty, high unemployment rates or a composite deprivation score may be most effective, as the largest differences (in the crude models) were seen for these deprivation measures.

## Conclusion

In this paper, we show that persons living in high-deprivation neighborhoods have significantly lower probability of participating in a population-based health check than those living in low-deprivation neighborhoods. This suggests the need to develop preventive health checks tailored to deprived neighborhoods (e.g. increasing incentives). Such studies will help answer if health checks, which are designed to meet the needs of residents in deprived neighborhoods will increase participation and prove to be effective in preventing disease.
